# Performance reporting for consumers: issues for the Australian private hospital sector

**DOI:** 10.1186/1743-8462-4-5

**Published:** 2007-05-30

**Authors:** Margo Sheahan, Russ Little, Sandra G Leggat

**Affiliations:** 1 Doctor of Public Health candidate, La Trobe University, Bundoora, Victoria 3086, Australia; 2 Consumer, Epworth Hospital, Melbourne, Victoria 3121, Australia; 3 Professor Health Services Management, School of Public Health La Trobe University, Victoria 3086, Australia

## Abstract

A group of consumers of private hospital services and their carers collaborated with staff of a Melbourne private hospital and with industry representatives to develop a consumer-driven performance report on cardiac services. During the development process participating consumers identified situational and structural barriers to their right to be informed of costs, to choice and to quality care. Their growing appreciation of these barriers led them to a different perspective on performance reporting, which resulted in their redirecting the project. The consumer participants no longer wanted a performance report that provided comparative quantitative data. Instead they designed a report that outlined the structures, systems and processes the hospital had in place to address the quality and safety of services provided. In addition, consumer participants developed a decision support tool for consumers to use in navigating the private health care sector. The journey of these consumers in creating a consumer driven performance report for a private hospital service may assist those responsible for governance of Australia's health system in choosing appropriate strategies and mechanisms to enhance private hospital accountability. The situational and institutional industry barriers to choice, information and quality identified by these consumers need to be addressed before public performance reporting for private hospitals is introduced in Australia.

## Background

The private health sector contributes significantly to Australia's health care system. In 2003–2004, private hospitals provided 33.3% of national total beds and 38.6% of the 6.84 million patient separations reported nationally [1]. Comprising health insurance, medical practitioners and hospital providers, the private health sector is regulated through a range of legislation that is administered by both state and national level organisations. Privately insured consumers seek benefits above public sector services such as choice of doctor and hospital and timing of procedure [2]. There is no coordinating body for the sector and consumers must choose among services offered by health insurers, doctors and hospital providers. Ensuring that the consumer is adequately informed of options and costs of treatment is an industry challenge [3].

The Australian Government Department of Health and Ageing (the sponsor) established the Consumer and Provider Partnerships in Health (CAPPS) program in early 2000 to increase consumer participation in health care [4]. In 2001 two of these CAPPS grants were made available to private hospitals. This paper describes one of the CAPPS private hospital projects; the development of a consumer driven performance report at the Epworth Hospital, then a 500 bed not-for-profit private hospital in Melbourne Australia. This project aimed to test a consumer driven private hospital performance report.

### Hospital performance reporting

There are three main reasons for public reporting on performance information [5]: supporting consumer choice [6,7], enabling accountability [7,8], and promoting quality [8-10]. Supporting consumer choice has been a particular focus in the United States, while accountability has been the focus in the United Kingdom [11] and Canada [5]. Accountability has also been the focus of public sector reports published by Australian State Governments that provide comparative data for public hospitals [12]. Use of performance reporting to promote quality is evident in many countries [5] and public reporting to improve both quality and accountability has become a priority in Australia in the wake of research [13,14] and a number of public inquiries [15-20] that highlighted the need for a safer and more accountable health care system [21-24].

Despite the identified need for performance reporting, there has been limited performance information available to consumers of Australian private health care. One of the limitations has been that Australia does not have a trusted national body to collect, collate and risk-adjust comparable hospital and doctor performance data [25] and without a trustworthy source, data are unlikely to be used to guide decision-making [26]. Only recently has the National Government required health insurers to publish information about private health insurance products in a way that enables consumers to compare products [27]. From 2007 the Private Health Insurance Ombudsman will publish data to enable consumers to compare products.

In Australia two frameworks for performance reporting have been described; public release information and purposeful reporting to consumers [11]. Public release information aims to fulfil a provider's duty to disclose pertinent information to the public and by so doing improve the accountability of the health system to citizens [7]. Hospital performance reports in Australia have typically employed a public release framework, focused on quality and accountability managed by a central government body [28].

In contrast purposeful reporting is tailored to the particular needs of consumer groups and specific decisions such as choice of hospital or doctor [11]. Purposeful reporting aims to promote public accountability by informing consumer choice enabling consumers to make informed decisions, assess quality and contribute to quality enhancement [29]. Unlike the public release method, the purposeful reporting approach works with consumers in constructing the report. This partnership guides 'knowledge construction' rather than 'information telling'.

Cognitive science research has shown that the knowledge construction approach provides greater support for the consumer's basic decision making process [29]. Simply, it has been shown that consumers are more likely to find a performance report useful when information relevant to their needs is made available in the report [30,31]. Given the lack of an industry structure to support public reporting, the purposeful reporting framework would be preferable to a public release method because consumers are actively involved in directing it. In addition this framework is aligned with the private sector's commitment to choice and provides principles to guide negotiation. For these reasons, the purposeful reporting method was expected to deliver more effective performance reporting for consumer decision making.

Consumer choice requires private health insurers to communicate the relative benefits of their health insurance products [27], and private hospitals and doctors to provide information about treatment costs [3]. Further, comparative information on hospital and doctor performance would assist consumer choice of hospital and doctor, at least in non-emergency situations [25]. To explore consumer needs for information on products, costs and performance this project engaged a group of consumers and their carers in the development of a performance report for private hospital cardiac services.

## Methods

### Setting

Epworth Hospital is a private not-for-profit hospital located in Melbourne, Australia. At the time of the project the hospital provided cardiac services to approximately 3,600 patients each year through a 58 bed cardiac facility supported by a large and well-equipped intensive care unit.

### Participants

Epworth partnered with two consumer groups to source participants for this project. Heartbeat Epworth Inc. represented consumers of acute cardiac surgical services and the Cardiomyopathy Association of Australia Ltd. represented consumers of acute cardiac medical services. Representatives from these organisations, including consumers and their carers, were invited to participate in the project from the time of proposal development through to completion of the project.

### Process

A steering committee was established comprising industry stakeholders and consumer representatives and met six times over the life of the 12 month project. The industry stakeholders represented health insurance, specialist and general medical practitioners and hospital management. Consumer members included the presidents and other members of the two self help consumer groups. An experienced consumer representative from an independent, not-for-profit organisation that promotes consumer perspectives in the Australian health system through policy research and consumer advocacy also participated. An expert conciliator familiar with the health industry was invited to chair so that power differentials of those on the steering committee would be addressed.

Four exploratory focus groups were held to explore consumer and carer (n = 51) perceptions of cardiac services provided by Epworth. Two of the focus groups focused on cardiac surgical services and two captured the perceptions of consumers of invasive cardiology services. It was important to separate surgery and cardiology as the two forms of treatment result in different consumer experiences. Focus group participants were invited by individual letter, through a notice in each partner association's newsletter and invitations to individual patients following their rehabilitation program.

Of the 51 initial focus group participants the average age was 71 years, 62% were male and 64% had been patients and the others carers. Participants had been involved in a range of cardiac related episodes spanning the past two decades. Interventions included; coronary artery bypass grafts, angioplasty and stenting, valve replacement, angiography and pacemaker implantation. Some members had experienced several admissions and the average length of stay for the participant group was 7 days. The demographics of the focus group participants reflected the general hospital cardiac patient population.

A question sheet, developed by the facilitator and project leader prior to the focus groups, was used to guide discussion. During these focus groups participants were introduced to the project aims to develop a performance report for cardiac services at Epworth Hospital and the structure and scope of the project. A project team that was answerable to the steering committee and facilitated by the project leader obtained a set of potential cardiac service performance measures and also examples of performance reports including a magazine that provided evaluative and comparative information for consumers. These were provided to help participants visualise different report formats. During the focus groups the participants identified and defined consumer quality issues and selected a report format that was meaningful to them. The sessions were taped and analysed by the facilitator. Focus group outcomes were agreed to by consumers in the steering committee and used to develop the performance report format and content.

Twenty-one participants and carers returned to participate in focus groups in the final four months of the project. It was unfortunate that less than half of the consumer participants were able to continue to attend the focus groups, but the expected and unexpected health and other issues made it difficult for some of the consumers and carers to continue to participate. These focus groups were provided with an opportunity to work with an expert editor of education textbooks and a graphic designer to develop the draft report. In these focus groups it was expected that the participants would agree on a performance report. In working towards this goal the participants prioritised 64 draft measures; relating to access (for example time from admission to angiogram); quality of care, (for example satisfaction with communication from their doctor and satisfaction with nursing skill); and discharge management (for example consumer confidence going home) [32]. However as the consumer participants explored these measures they began to question the organisational and private health system processes and structures.

At this point in time, approximately 10 months into the 12-month project, the consumers wanted to redirect the project. They no longer wanted comparative quantitative data on performance; instead they indicated that they would prefer information on the processes and structures employed by the hospital to ensure its standard of care. In addition the participants sought information on processes and structures that shaped their access to quality care. The project leader responded to this consumer voice and negotiated with the steering committee and the sponsor to change the focus of the project to accommodate the new-found awareness of the consumer participants.

## Results and discussion

During the project the consumer participants became increasingly aware of existing structural barriers to reliable performance reporting in the Australian private hospital sector. The consumers discussed factors that limited the amount of information they received on health care costs, that reduced their ability to make choices and that impacted the quality of care. As participants became more aware of these barriers they sought ways to address them, changing the direction of the project. This resulted in a substantial change to the format of the planned performance report and to the development of a decision support tool to help consumers navigate the private health care industry.

### Barriers to information on costs

Consumers have a recognised right to be informed of the cost implications of a hospital admission [3,33,34]. However the consumer participants of this project voiced concerns about an industry failure to meet this right. Some participants noted that they did not realise until after their admission that their health insurance did not cover the 'throw away tubes and things' used in intensive care [32]. The participants also became aware that some insurance products excluded particular services. They were concerned that insurance companies could sell insurance products that excluded emergency cardiac services such as 'stenting'. Indeed one participant won group support when he argued that product exclusions for any emergency service was unconscionable, reasoning that, "*I cannot get house insurance and not have the house completely insured."*

Participants agreed that life saving services needed in a time critical situation should not be excluded from health insurance products. The participants reflected that usually the insurance purchase decision was made quite separately to a decision to access private health care and there was a high risk that the consumer would not link health insurance purchase with a judgement about their likely health service needs. This failure to link relevant information was made worse by the fact that there is typically a considerable period of time between the purchase of private health insurance and its use when accessing health care. This observation led participants to the view that consumers of private health insurance should be reminded annually of their cover, just as one is for house and car insurance. The focus group participants then noted that none of them could recall ever receiving such a communication from their health insurance provider.

The participants discussed their experiences trying to obtain information from health care practitioners – individual clinician or team information is not collected and is certainly not made easily available to consumers. For example the consumers complained that lack of information about costs arose because the surgeon may not be able to advise the consumer of the expected anaesthetic fee. A participant asked, "*He [surgeon] selects the anaesthetist but says I don't know what it will cost..... How can you cope with that type of stuff*?"

Participants realised that barriers to their right to be informed were structural in nature and highlighted the lack of collaboration between the health funds, the doctors and the hospitals. This resulted in consumer participants questioning whether a report, which provided comparative data, would be reliable, or even useful. With this shared awareness, the participants began to seek strategies to raise consumer awareness of the cost implications of private health services.

### Barriers to choice

One of the rationales for performance reporting is that it facilitates consumer choice [6,7]. However the participants in this project hotly challenged the idea that they had choice.

How many people do have choice? I followed the track, and just ended up here

*You have no choice really. You put your life in your doctor's hands. The alternative is – you are dead. You are not in the mood to agree or choose*.

In Australia choice of doctor, hospital and timing of procedure is a promoted benefit of private health cover. However the consumers of cardiac services in this project indicated that situational barriers, such as needing emergency access to services, often limited their ability to exercise choice.

The consumers also recognised that their ability to exercise choice was often limited by structural barriers as well. Participants acknowledged that referral networks had impacted their navigation of the private health care system. One participant described how annoyed he was when his general practitioner had sent him to the 'closest' specialist, rather than 'the best' [32]. While they agreed that consumers needed to be more 'up front' with their doctors about their priorities to influence traditional referring practices, the group conceded that traditional doctor referral practices and well known communication issues may still undermine the consumer choice that is promoted in the private health sector. For these reasons consumers felt that it was unrealistic to assume that the provision of a performance report for consumers of cardiac services would be sufficient to promote choice without structural changes to the private health care system.

### Barriers to consumer interests in quality

The consumer participants reflected on other structural barriers that they believed limited the capacity of the industry to coordinate relationships between industry stakeholders in the interests of safety and quality of care. Doctors were seen as independent practitioners and their cooperation with and contribution to hospital quality activities was optional. One participant said;

*The problem is doctors are not employed by the hospital. The hospital hasn't got that much control over the situation*.

For this reason, instead of quantitative data on quality and safety which the participants felt might not be reliable, the participants wanted the report to demonstrate the processes and structures through which the hospital managed the quality of medical care provided. The consumers wanted particular information on how the hospital ensured quality and safety given its limited capacity to impose medical governance.

The capacity of the hospital to deliver on quality was also questioned due to a perceived structural tension between hospitals and health insurance providers. The consumers had come to realise that the care they received was not only based on their clinical need, but was also influenced by the arrangements between their health insurance provider and the hospitals and clinicians.

*The hospital – provider agreements create tension on the hospital between the needs of the patient and needs of the hospital. I was told I could go home and I asked to stay longer. They did the calculations and said it was OK. It becomes uneconomical for hospitals to keep patients. We need to know more, in the interests of transparency*.

These participant concerns about the structural barriers to safety and quality of care raised questions of whether a performance report would be useful in promoting accountability.

### Revised performance report

It was anticipated that the cardiac performance report would facilitate consumer choice by providing data on access, effectiveness, communication and participation, care, continuity of care, human needs and efficiency [11]. However these dimensions did not meet the information needs of the consumers once they had identified the situational and structural barriers to information, choice and quality care. When asked to explain the radical shift from their earlier position, the consumers explained that as they became more aware of the data to be presented, it became clear to them that unless it was provided by an independent trusted source it would not be reliable. They decided that it was naive to believe that comparative data would guide choice in an industry that lacked an independent process for collection, collation, reporting and interpretation of data. As one participant said; *"data is like a bikini, what it reveals is interesting but what it conceals is vital" *[32]. This led to a preference for qualitative data on organisational processes. It became apparent that given there was no trusted coordinating body these consumers trusted data they could validate from their own experience.

The outcome was a seven page performance report that outlined the structures and processes by which the hospital sought to provide quality care, including information on accreditation, medical governance, the nursing model, infection control and medication safety. For example, the section on medication safety provided an opportunity for consumers to play a role in reducing the incidence of medication errors, one of the target areas for risk management in Australian health care [35]. The report also provided information on services with which the patient could engage to enhance their care, such as discharge planning, conciliation process and services linked to informed financial consent. Following the project the performance report was distributed to patients admitted to the cardiac units to facilitate their participation in their care.

These consumer driven changes challenged common understandings of performance reports as a vehicle for providing comparative data on which consumers may base an informed choice. Indeed the performance report the consumers requested moved beyond an individual focus and became a tool to engage the consumer not only in their care but also in improving hospital processes. The purposeful reporting approach that was employed in this project supported such a move, as the purposeful reporting principles encouraged the involvement of consumers in developing the report, shaping both its content and format. Secondly, the purposeful reporting approach supported strategies to increase consumer involvement in their care and to improve hospital processes [11].

### The decision support tool

The consumer participants argued that given the situational and structural barriers quantitative comparative performance reports would not help consumers access, organise and apply information provided by the different service providers in the private health sector. Instead the consumers decided they should develop a decision support tool to assist other consumers and carers to navigate the private health sector from the time of health insurance purchase through to health service use. The decision support tool was designed to guide consumers through choice of health fund product, doctor, and hospital. For example, the decision tool highlighted health insurance product exclusions, referred consumers to each health fund's 'Key Features Guide' to enable comparison of different products and provided information about ambulance service policies. In 2003 the decision tool was presented to the existing private health sector peak quality and safety organisation in the hope of obtaining funding for national distribution, but the tool was not funded.

### Negotiating consumer voice

The purposeful reporting framework facilitated consumer ownership and control and enabled individual consumers to move beyond their particular interests and act to improve the situation for others. The steering committee provided an opportunity for consumers and carers to collaborate with stakeholders to provide the consumers with a voice at higher-level decision making. However, redirection of the project from a cardiac performance report comprising comparative data may arguably have aligned with hospital and industry interests, and raises questions about whether power distorted negotiations and consumer voice. The transparency and accountability of the steering committee process, with the minutes, range of members and independent expert chair, and the transparent negotiations with the sponsor, played a key role in building confidence that the decision to redirect the project was indeed consumer driven [4]. Given these checks and balances, it was concluded this partnership had facilitated a power shift from stakeholders and the project funding body to consumers [4].

As participants articulated concerns about industry barriers to choice, accountability and quality, the project was redirected. First the consumers realised, in support of findings from the United States [26], that the lack of a trusted coordinating body rendered comparative quantitative data untrustworthy as an accountability tool. Second as the consumer participants shared their care experiences they identified structural barriers to cooperative information sharing, to their ability to make choices and to quality of care initiatives. The consumers perceived that these barriers also lessened the usefulness of a quantitative comparative data-driven performance report for their purposes. This resulted in the change in focus of the cardiac performance report and development of a decision support tool to assist consumers choose among the services offered by health insurers, doctors and hospital providers when seeking to access private health care services. To date the decision tool remains untested in the industry suggesting limited attention to how consumer voice may increase accountability.

## Conclusion

The journey of these consumers in creating a consumer driven performance report for a private hospital service may assist those responsible for governance of Australia's health system in choosing appropriate strategies and mechanisms to enhance private hospital accountability. The situational and institutional industry barriers to choice, information and quality identified by these consumers need to be addressed before public performance reporting for private hospitals is introduced in Australia. This study has highlighted issues in public performance reporting for private hospital services and suggests the need for further research to assist in the development of effective and acceptable accountability strategies and mechanisms for health markets in liberal democracies.

## Authors' contributions

I confirm that all authors have read and approved the manuscript.
